# Antimigraine drug, zolmitriptan, inhibits high-voltage activated calcium currents in a population of acutely dissociated rat trigeminal sensory neurons

**DOI:** 10.1186/1744-8069-2-10

**Published:** 2006-03-20

**Authors:** Tomoko Morikawa, Yoshiyasu Matsuzawa, Koshi Makita, Yoshifumi Katayama

**Affiliations:** 1 Department of Anesthesiology, Graduate School of Medicine, Tokyo Medical and Dental University, Tokyo, Japan; 2 Department of Autonomic Physiology, Medical Research Institute, Tokyo Medical and Dental University, Tokyo, Japan

## Abstract

**Background:**

Triptans, 5-HT_1B/ID _agonists, act on peripheral and/or central terminals of trigeminal ganglion neurons (TGNs) and inhibit the release of neurotransmitters to second-order neurons, which is considered as one of key mechanisms for pain relief by triptans as antimigraine drugs. Although high-voltage activated (HVA) Ca^2+ ^channels contribute to the release of neurotransmitters from TGNs, electrical actions of triptans on the HVA Ca^2+ ^channels are not yet documented.

**Results:**

In the present study, actions of zolmitriptan, one of triptans, were examined on the HVA Ca^2+ ^channels in acutely dissociated rat TGNs, by using whole-cell patch recording of Ba^2+ ^currents (I_Ba_) passing through Ca^2+ ^channels. Zolmitriptan (0.1–100 μM) reduced the size of I_Ba _in a concentration-dependent manner. This zolmitriptan-induced inhibitory action was blocked by GR127935, a 5-HT_1B/1D _antagonist, and by overnight pretreatment with pertussis toxin (PTX). P/Q-type Ca^2+ ^channel blockers inhibited the inhibitory action of zolmitriptan on I_Ba_, compared to N- and L-type blockers, and R-type blocker did, compared to L-type blocker, respectively (p < 0.05). All of the present results indicated that zolmitriptan inhibited HVA P/Q- and possibly R-type channels by activating the 5-HT_1B/1D _receptor linked to G_i/o _pathway.

**Conclusion:**

It is concluded that this zolmitriptan inhibition of HVA Ca^2+ ^channels may explain the reduction in the release of neurotransmitters including CGRP, possibly leading to antimigraine effects of zolmitriptan.

## Background

It is known that the pain associated with migraine is relieved by triptans, 5HT_1B/1D _agonists, including sumatriptan, zolmitriptan, naratriptan and so on. Indeed, they are in clinical use for treatment of migraine. It is shown that trigeminal ganglion stimulation leads to the release of CGRP in humans and cats, which is antagonized by sumatriptan administration [1]. Subsequently, several lines of histochemical and electrophysiological studies demonstrate the involvement of 5HT_1B/1D _agonist in neurotransmitter release from trigeminal ganglion neurons (TGNs). First, 5HT_1B _and/or _1D _receptors are localized in trigeminal vascular systems [2]. 5HT_1B _receptors are demonstrated on dural arteries [2] and 5HT_1D _receptors on trigeminal sensory neurons including peripheral and central projections [2-4]. Second, small and medium- sized TGNs possess 5HT_1B/1D _receptors, colocalized with CGRP and Substance P [5]. Third, naratriptan inhibits neuronal activity in TGNs [6]. Fourth, synaptic transmission from TGNs to central trigeminovascular neurons is blocked by activation of presynaptic 5HT_1B/1D _receptors on central terminals of meningeal nociceptors [7]. All of these studies suggest that triptans might act on 5HT_1B/1D _receptors of TGNs and inhibit the release of neurotransmitters such as CGRP, reducing central and/or peripheral neuronal excitability.

An activation of high-voltage activated (HVA) Ca^2+ ^channels is known to trigger the release of neurotransmitters and to control numerous neuronal functions such as neuronal excitability. HVA Ca^2+ ^channels are divided into four subtypes; that is N-, P/Q-, L-, and R-type channels. All of four subtypes of HVA Ca^2+ ^channels are demonstrated to be expressed in TGNs [8]. Recent findings indicate that the blockade of HVA Ca^2+ ^channels prevents CGRP release and prevents dural vessel dilation, and so HVA Ca^2+ ^blockade might minimize neurological inflammation [9]. Although it is shown that N- and P/Q-currents are inhibited via G protein-coupled mechanisms by agonists for 5HT_1A _and _1D _receptors in the primary spinal neurons of Xenopus larvae [10,11], effects of 5HT_1B/!D _agonists on HVA Ca^2+ ^channels in mammalian TGNs have not yet been evaluated.

As mentioned above, involvement of triptans in modulation of CGRP release as well as neuronal activity in the trigeminal ganglion is highly plausible. This prompted us to examine whether or not triptans could act on HVA Ca^2+ ^channels of TGNs, leading to inhibition of the release of CGRP and neurotransmission, possibly involved in generation of migraine. In the present study, electrophysiological experiments were undertaken to analyze actions of zolmitriptan, one of triptans, on HVA Ca^2+ ^channels using cultured neonatal rat TGNs. This paper clarified that zolmitriptan could inhibit HVA Ca^2+ ^channels by activating 5HT_1B/1D _receptor coupled to G_i/o _pathway.

## Results

Currents carried by Ba^2+ ^passing through HVA Ca^2+ ^channels, I_Ba_, were recorded from somata of neonatal rat TGNs, small to medium size of 22 to 27 μm in diameter. The peak amplitude of I_Ba _in control varied within the range from 230 to 1200 pA (mean ± S.E.M.; 508.5 ± 31.0 pA, n = 37).

### Concentration-dependent action of zolmitriptan on I_Ba_

**Figure 1:**
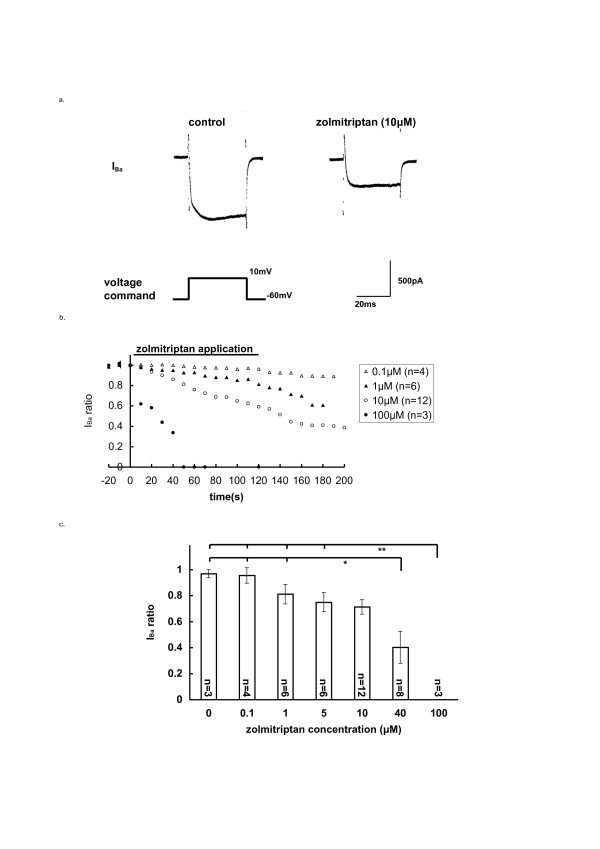
**Inhibition of HVA I_Ba _by zolmitriptan**. (a) Typical illustration of I_Ba _elicited in response to command pulses from -60 mV to 10 mV for 40 ms. I_Ba _was inhibited by 2 min application of 10 μM zolmitriptan. (b) The average time course of I_Ba _inhibition by zolmitriptan at four different concentrations. Superfusing application of zolmitriptan started at t = 0 and lasted for 120 s during the period indicated by horizontal bar. Mean value of the relative amplitude of I_Ba _compared to the control I_Ba _at t = 0 was plotted on ordinate (I_Ba _ratio) against time on abscissa. The number of neurons examined is indicated for the respective concentrations. S.E.M. value was not indicated. (c) Concentration-inhibition relationship for zolmitriptan. Bar graph shows the relative amplitude of I_Ba _at two minutes after application of zolmitriptan compared to the control. (*p < 0.05 **p < 0.01).

Zolmitriptan was applied to TGNs by superfusion for two minutes. As shown in Fig. 1a, I_Ba _was inhibited in the presence of zolmitriptan at 10 μM. Inhibitory actions of zolmitriptan on I_Ba _were examined at concentrations between 0.1 and 100 μM (Fig. 1b, the number of cells indicated). Zolmitriptan at lower concentrations slowly started depressing the I_Ba _at 10 to 20 s from the onset of application. This depressing action slowly increased but could not reach its maximum in 2 min at concentrations lower than 10 μM. On the other hand, at 100 μM, the I_Ba _was very rapidly inhibited within 10 s and completely abolished within one min of the drug application.

As noticed from Fig. 1b, this inhibitory effect of zolmitriptan on I_Ba _lasted after the end of the drug application and afterwards became more marked, attaining to its peak. Then, it should be noted that the inhibitory action of zolmitriptan on I_Ba _could be hardly washed out. Therefore, the inhibitory effect of the drug was compared by using the I_Ba _ratio (see Method and figure legend) at 2 min after the onset of the application. The I_Ba _ratios were 0.96 ± 0.06 (0.1 μM, n = 4), 0.81 ± 0.08 (1 μM, n = 6), 0.75 ± 0.07 (5 μM, n = 6), 0.71 ± 0.06 (10 μM, n = 12), 0.40 ± 0.12 (40 μM, n = 8), and 0.00 ± 0.00 (100 μM, n = 3), and compared with the I_Ba _ratio of control group without zolmitriptan (0.97 ± 0.03, n = 3), as summarised in Fig. 1c, showing the concentration-inhibition relationship for the action of zolmitriptan on I_Ba_.

### Action of zolmitriptan, inhibited by a 5HT_1B/1D _antagonist

**Figure 2:**
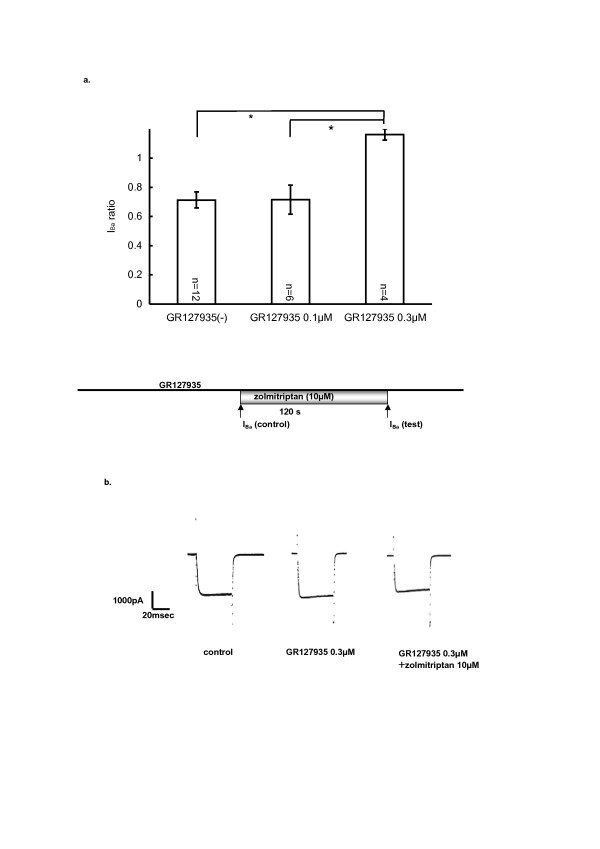
**GR127935 modulation on zolmitriptan-sensitive I_Ba_**. (a) GR127935, 5HT_1B/1D _antagonist, depressed the inhibition of I_Ba _by zolmitriptan (10 μM). Bar graph shows that the zolmitriptan-induced inhibition of I_Ba _was significantly reduced by pretreatment with 0.3 μM of the antagonist GR127935 (*p < 0.05). I_Ba _ratio is the value that I_Ba _(test) was divided by I_Ba _(control). Inset shows the time course of GR127935 and zolmitriptan application. I_Ba _(control) and I_Ba _(test) were recorded as shown in the chart. (b) Typical illustration of I_Ba _in control (left), in the presence of 0.3 μM GR127935 (center), and 10 μM zolmitriptan added on 0.3 μM GR127935 (right).

Since triptans are known to act as 5-HT_1B/1D _agonists, we examined whether or not the zolmitriptan-induced inhibition on I_Ba _could be blocked by a 5-HT_1B/1D _receptor antagonist, GR127935. The preparations were pretreated with GR127935 for 2 min; no direct actions of the antagonist on I_Ba _were observed at 0.3 μM. Following GR127935 application for more than 2 min, zolmitriptan (5 and 10 μM) was added to the superfusate. The I_Ba _ratios with 10 μM zolmitriptan were 0.71 ± 0.06 (without GR127935, n = 12), 0.72 ± 0.10 (0.1 μM GR127935, n = 6), 1.10 ± 0.04 (0.3 μM GR127935, n = 4), as summarized in Fig. 2. It was shown that the zolmitriptan-induced inhibition of I_Ba _was significantly reduced by GR127935 at 0.3 μM. On the other hand, the I_Ba _ratios with 5 μM zolmitriptan were 0.75 ± 0.07 (without GR127935, n = 6), 0.84 ± 0.13 (0.1 μM GR127935, n = 4), showing no significant inhibition. These data suggested that zolmitriptan inhibited I_Ba _by activating 5-HT_1B/1D _receptors. It should be added that GR127935 at concentrations higher than 1 μM occasionally inhibited I_Ba_.

### Action of zolmitriptan, mediated by G-protein pathway

**Figure 3:**
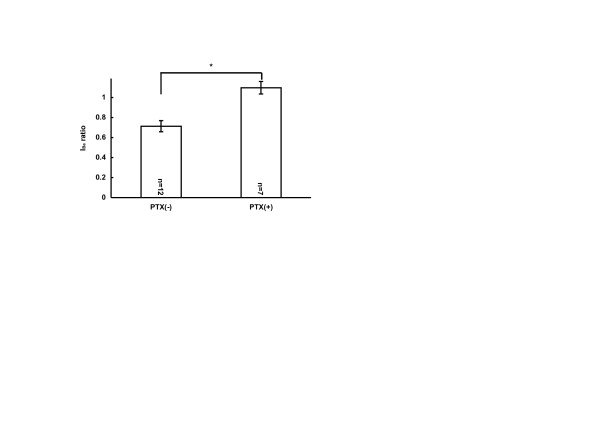
**PTX modulation on zolmitriptan-sensitive I_Ba_**. PTX treatment prevented the inhibition of I_Ba _by zolmitriptan (10 μM). Bar graph shows that the zolmitriptan-induced inhibition of I_Ba _was significantly reduced by overnight treatment of 500 ng/ml PTX (*p < 0.05). Recording of I_Ba _(control) and I_Ba _(test) in the presence of zolmitriptan were made according to the same time course shown in the inset of figure 2. I_Ba _ratio means I_Ba _(test)/I_Ba _(control).

It is widely accepted that some of 5-HT receptor subtypes are G-protein coupled. Possible involvement of G-protein pathways in the present action of zolimitriptan was tested by using pertussis toxin (PTX, an irreversible inhibitor of G_i/o _proteins). When cultured TGNs were treated overnight with PTX (500 ng/ml), zolmitriptan at 10 μM could not exert an inhibitory effect on I_Ba_; the amplitude of I_Ba _in control was almost the same as that of I_Ba _in the presence of zolmitriptan; that is I_Ba _ratio = 1.0 as shown in Fig. 3. Thus, PTX pretreatment prevented the inhibition of I_Ba _by zolmitriptan, while I_Ba _was depressed by zolmitriptan without the PTX pretreatment. This significant change induced by PTX indicated the role of G-proteins in the zolmitriptan inhibition of I_Ba_.

### Pharmacological profile of I_Ba_, sensitive to zolmitriptan

Characteristics of I_Ba _inhibited by zolmitriptan were pharmacologically determined by using a variety of selective Ca^2+ ^channel blockers. Indeed, four types of HVA Ca^2+ ^channels are known to be expressed in TGNs; that is, N-type, P/Q-type, R-type, and L-type channels. In the present experiments, therefore, ω-conotoxin GVIA (ω-CgTx, 1 μM), ω-agatoxin IVA (ω-Aga, 0.2 μM), SNX-482 (0.1 μM), and nicardipine (10 μM) were used to examine possible contribution of each Ca^2+ ^channel to the zolmitriptan-sensitive I_Ba_, respectively. It is confirmed that all four Ca^2+ ^blockers reduced I_Ba_; ratios of I_Ba _in the presence of Ca^2+ ^blockers to control I_Ba _were 0.42 ± 0.05 (ω-CgTx, n = 5); 0.58 ± 0.04 (ω-Aga, n = 4); 0.84 ± 0.05 (SNX-482, n = 7); and 0.43 ± 0.08 (nicardipine, n = 4).

**Figure 4:**
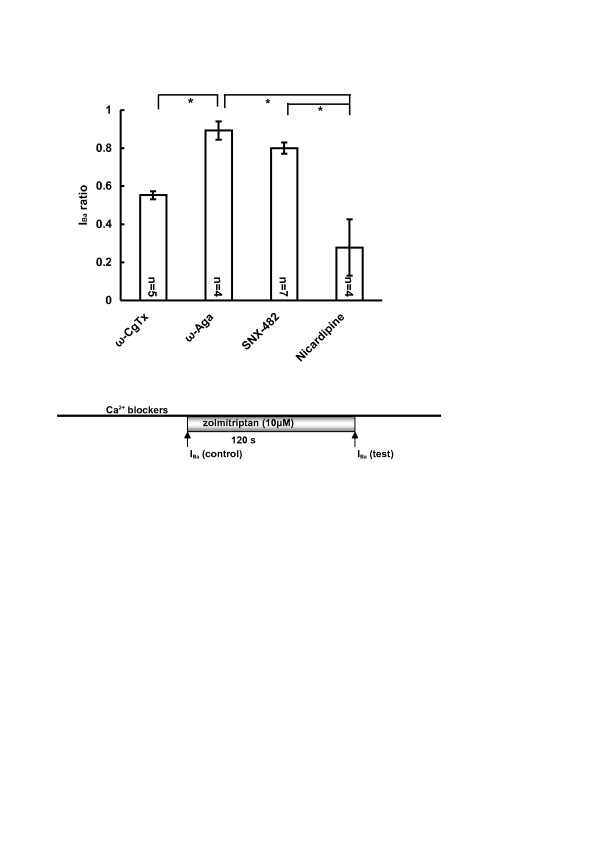
**Pharmacological characteristics of zolmitriptan-sensitive I_Ba_**. Bar graph shows that inhibition of I_Ba _was significantly reduced with ω-Aga, compared to those with ω-CgTx and nicardipine, Aand with SNX-482, compared to that with nicardipine (*p < 0.05). I_Ba _(control) after pretreatment with Ca^2+ ^blockers and I_Ba _(test) 2 min after adding zolmitriptan were recorded as indicated in the inset. I_Ba _ratio was obtained by I_Ba _(test)/I_Ba _(control).

After pretreatment with each of blockers for 2 min, zolmitriptan (10 μM) was added to the superfusing solutions, and I_Ba _ratios were obtained (see inset of Fig. 4). When pretreated with ω-CgTx, the I_Ba _ratio was 0.55 ± 0.02 (n = 5); with ω-Aga, 0.89 ± 0.05 (n = 4); with SNX-482, 0.80 ± 0.03 (n = 7); and with nicardipine, 0.28 ± 0.15 (n = 4) (Fig. 4). The I_Ba _ratios after pretreatment with ω-Aga or SNX-482 seemed to be larger than the ratio 0.71 ± 0.06 (10 μM zolmitriptan without Ca^2+ ^blockers) in Fig. 1c, suggesting a possibility that Ca^2+ ^channels sensitive to ω-Aga or SNX-482 likely contributed to the blockade of zolmitriptan I_Ba _inhibition. Indeed, significant difference was detected between ω-Aga and ω-CgTx or nicardipine, and also between SNX-482 and nicardipine (Fig. 4), indicating that blockade of P/Q-type and R-type Ca^2+ ^channels with ω-Aga and SNX-482 reduced the inhibition of I_Ba _by zolmitriptan. Therefore, it is likely that P/Q-type and R-type channels could be inhibited by zolmitriptan by acting on 5-HT_1B/1D _receptors through G proteins pathways.

## Discussion

The present experiments demonstrated modulating actions by zolmitriptan on I_Ba _of the rat isolated TGNs. Zolmitriptan inhibited HVA Ca^2+ ^currents carried by Ba^2+ ^in a concentration-dependent manner within the concentration range between 0.1 μM and 100 μM by acting on 5HT_1B/1D _receptor through G_i/o _protein-coupled pathway.

5HT receptors are divided into 7 families, 5HT_1~7 _receptors, on the basis of their amino acid sequences and other properties. 5HT_1 _receptors are further subdivided according to their physiological functions, binding affinity and other features [12]. The present study showed that GR127935, a potent 5HT_1B/1D _receptor antagonist abolished the effect of zolmitriptan, meaning that zolmitriptan acted on 5HT_1B/1D _receptor.

5HT_1B _and/or _1D _subtypes are known as G-protein mediated receptors. In the present study, pretreatment with PTX inhibited the I_Ba _inhibition by zolmitriptan, indicating the involvement of G_i/o _protein coupled pathway. This observation might be compatible with the previous reports that an increase in intracellular Ca^2+ ^level by 5HT_1 _receptor is associated with activation of G_i_/G_o _protein coupled pathway [13,14] and that the modulation of neuronal voltage-gated Ca^2+ ^channel is mediated by receptors coupled to PTX-sensitive G proteins [15,16]. In this context, possible involvement of stimulatory of G-proteins (G_s_) in the zolmitriptan action should be further investigated by using cholera toxin. A recent report shows that sumatriptan could activate the other second messenger MAPK pathway leading to changes in intracellular Ca^2+ ^changes [17]. This possibility for the action of zolmitriptan remains to be considered in future.

It is reported that triptans, antimigraine drugs might inhibit the release of vasoactive neuropeptide from trigeminovascular nerve endings and also inhibit transmission of nociceptive impulses to second-order neurons of the trigeminocervical complex, resulting in the antimigraine effect of triptan [18]. It is known that the trigeminal ganglion possesses small to medium size 5HT_1B/1D _receptor positive peptidergic neurons [4,5] and furthermore that antimigraine drugs could block synaptic transmission between meningeal nociceptors and central trigeminal neurons presynaptically [7]. All of these suggest that HVA Ca^2+ ^channels, highly responsible to neurotransmitter release from presynaptic terminal, might be involved in the antimigraine effects of triptans. Indeed the present study showed that HVA I_Ba _of TGNs was affected by zolmitriptan, a 5HT_1B/1D _agonist, strongly advocating the idea that triptans inhibited neurotransmitter release from peripheral or central presynaptic terminal through HVA Ca^2+ ^channels.

It is important to determine which subtypes of HVA Ca^2+ ^channels might essentially contribute to the release of different neurotransmitters from various classes of neurons. Some paper mentioned simply about HVA Ca^2+ ^subtype on trigeminal neurons, but there is no consensus about which subtypes mainly contribute yet. Ebersberger et al shows that discharge patterns of trigeminal second order neurons with dural input are different in the presence of each HVA Ca^2+ ^subtype blockade [19], On the other hand, Hong et al showed that N- and P/Q-channels are important for the release of CGRP from perivascular TGNs [20] and the release of CGRP is shown to be prevented when N-, P/Q- or L- channels are blocked on trigeminal vascular neuron [9]. The present study demonstrated that the inhibition of zolmitriptan-sensitive I_Ba _in small-medium TGNs depended mainly on activation of P/Q- and R-type channels.

P/Q-type Ca^2+ ^channels are reported to locate in all brain structure [18] and also in the trigeminal ganglia [8]. Furthermore, α-eudesmol, a P/Q-type channel blocker, inhibits the release of a neuropeptide from perivascular trigeminal sensory nerves [21]. These observations may support our present findings that P/Q-type channels might be possible sites on which zolmitriptan could act in cultured neonatal rat TGNs. Although N-type is also known to locate in DRG neurons [22-24], a few studies show the N-type channel dominance in TGNs. The present study with ω-CgTx also could not statistically demonstrate an appreciable involvement of N-type channels in the inhibition of zolmitriptan-sensitive I_Ba _of cultured rat TGNs.

R-type Ca^2+ ^channels are shown to locate presynaptically in the central nervous system, but the transmitter release mediated by R-type channels is less efficient than that by P/Q-and N-type channels [25]. In the process of development, R-type channels are replaced by P/Q-type ones in the central synaptic transmission [26]. There are similar results for Ca^2+ ^channel subtypes obtained from neonatal and adult TGNs; in neonatal 4% are provided with P/Q-type while 15% with R-type one [8]; in adult 40% with P/Q-type while 5% to R-type [27]. In this context, the present study, for the first time, demonstrated possible involvement of R- as well as P/Q-type channels in the actions of zolmitriptan on the cultured neonatal rat TGNs.

Although zolmitriptan (0.1~100 μM) inhibited I_Ba _of cultured TGNs, it is difficult to determine the effective concentration of zolmitriptan acting in vivo on the trigeminal ganglion. Sumatriptan is reported to induce discharges in dural primary afferent neurons at concentrations between 0.24 and 24 μM [28] and also cause vasocontraction in rat isolated vena portae smooth muscle at concentrations between 0.001 and 10 μM [29]; these indicate that actions of two triptans could be exerted at similar concentrations.

## Conclusion

Zolmitriptan inhibited I_Ba _in a concentration-dependent manner by acting on 5HT_1B/1D _receptor. P/Q- and possibly R-type calcium channels contributed to the inhibition of I_Ba _by zolmitriptan. G_i/o _protein pathway were involved. Although this action of zolmitriptan on HVA Ca^2+ ^channels might explain the antimigraine effect, more detailed research of second messenger pathway would reveal the further mechanism leading to antinociceptive effect of triptans and pain pathway of migraine.

## Method

### Animal preparation

All procedures were carried out in accordance with the guidelines for Animal Experimentation in Tokyo Medical and Dental University (No.0060010). Wistar rats (0–7 days after birth, Saitama Experimental Animals Supply Inc., Japan) were anesthetized by pentobarbital (i.p.). After the decapitation of the rats, trigeminal ganglia were dissected and treated with papain (20.3 units/ml) in low- Ca^2+ ^and low-Mg^2+ ^Krebs' solution for 30 min at 37°C, washed with modified Krebs' solution and triturated using fire-polished Pasteur pipettes. Neurons were plated onto poly-L-lysine pretreated 35 mm dishes. The plating medium contained Dulbecco's modified Eagle's medium with10% calf serum. The TGNs were kept in culture in modified Krebs' solution saturated with 5% CO_2 _at 37°C for 2 hours to one day before experiment. The ionic composition of the modified Krebs' solution was (mM): NaCl, 117; KCl, 4.7; CaCl_2_, 2.5; MgCl_2_, 1; glucose, 11; 3-(*N*-morpholino) propanesulfonic acid (MOPS), 25; and pH 7.2 adjusted with NaOH. The low-Ca^2+ ^and low-Mg^2+ ^Krebs' solution was made by adding EDTA (2.5 mM) to the modified Krebs' solution.

### Electrophysiological recording

Membrane currents were recorded from somata of cultured TGNs in the whole-cell voltage clamp configuration of patch clamp technique with an Axopatch 1D amplifier (Axon Instrument). Currents were filtered low-pass at 2 Hz by the built-in Bessel filter, and recorded on a chart recorder (San-ei) for later analysis. Patch pipettes were pulled from borosilicate glass capillaries (Harvard) using a puller (Narishige co.), and had input resistance of 5–10 MΩ after polishing. The ionic composition of the patch pipette solution was (mM): CsCl, 100; MOPS, 40; MgCl_2_, 1; EGTA, 10; CaCl_2_, 1; ATP, 2 and pH 7.2 adjusted with KOH. A series resistance of the recording system was not electrically compensated.

Currents carried by Ba^2+ ^passing through HVA Ca^2+ ^channels, I_Ba_, were evoked by depolarizing voltage step command pulse to +10 mV for 40 ms from a holding potential of -60 mV every 10 s. For isolating Ba^2+ ^currents an external solution was used, containing (mM): TEA-Cl 140; CsCl, 2.5; BaCl_2_, 2.5; MgCl_2_, 1; Glu, 11; HEPES, 10 and pH 7.3 adjusted with TEA-OH. The amplitude of I_Ba _was determined as the difference between the baseline and the peak inward current during each command pulse.

External solutions were applied continuously via a polyethylene tube mounted on a micromanipulator and the tip of the tube was positioned within 10 mm of the recorded neurons. External solution was kept at 37°C.The capacity of chamber was 150 μl and the flow rate of solution was 2 ml/min.

### Materials

Zolmitriptan was a gift from Astrazeneca. Zolmitriptan was dissolved in dimethylsulfoxide (DMSO) and stored at -20°C. More dilute solutions were made daily dissolved in external solution before every experiment. ω-CgTx, ω-Aga and SNX-482 were purchased from Peptide Institute. Nicardipine was from Sigma. GR127935 was from Tocris.

### Data analysis

All data are expressed as means ± S.E.M. I_Ba _ratio of Fig. 1b was expressed as the relative amplitude in response to each step command pulse compared to control values, and I_Ba _ratios shown in Fig 1c, 2, 3, 4 were expressed as the relative amplitude after 120 s zolmitriptan application compared to control values in the absence of zolmitriptan. Statistical significance was assessed with Student's t-test for simple comparisons and Bonferroni-type multiple t-test for multiple comparison. Differences of P < 0.05 were considered to be significant.

## List of Abbreviation

TGN, trigeminal ganglion neuron; HVA, high-voltage activated; I_Ba_, Ba^2+ ^currents; CGRP, calcitonin gene-related peptide; PTX, pertussis toxin; ω-Aga, ω-agatoxin IVA; ω-CgTx, ω-conotoxin GVIA; DRG, dorsal root ganglion; i.p., intraperitoneally; MOPs, 3-(*N*-morpholino) propanesulfonic acid; EDTA, ethylenediaminetetraacetic acid; EGTA, ethylene glycol-bis(beta-aminoethyl ether)-N,N,N',N'-tetraacetic acid; HEPES, 2-[4-(2-Hydroxyethyl)-1-piperadinyl] ethansulfonic acid; DMSO, dimethylsulfoxide.

## Competing interests

The author(s) declare that they have no competing interests.

## Authors' contributions

T. Morikawa conceived of the study, participated in design of the study, carried out cell-culture and electrophysiological experiments, performed the statistical analysis, and prepared the manuscript as a main investigator. Y Matsuzawa participated in experiments and discussion. K Makita participated in design of the study and did the entire summary and discussion from the viewpoint of the pain clinic. Y Katayama conceived of the study, performed in design of the study, helped to prepare the manuscript and gave financial support of the present study and approval of this version to be published. All authors read and approved the final manuscript.
